# T Follicular Regulatory Cells: Choreographers of Productive Germinal Center Responses

**DOI:** 10.3389/fimmu.2021.679909

**Published:** 2021-06-10

**Authors:** Yisi Lu, Joe Craft

**Affiliations:** ^1^ Department of Immunobiology, Yale School of Medicine, New Haven, CT, United States; ^2^ Department of Internal Medicine, Yale School of Medicine, New Haven, CT, United States

**Keywords:** autoimmunity, B cell follicle, Bcl6, germinal center, humoral immunity, T follicular helper cells, T follicular regulatory cells

## Abstract

T follicular regulatory cells, or Tfr cells, are a discernable population of regulatory T (Treg) cells that migrate to the B cell follicle and germinal center (GC) upon immune challenge. These cells express the transcription factor Bcl6, the master regulator required for development and differentiation of T follicular helper cells, and are among a group of previously described Treg cells that use T helper cell–associated transcription factors to adapt their regulatory function to diverse milieus for maintenance of immune homeostasis. While there is consensus that Tfr cells control B-cell autoreactivity, it has been unclear whether they regulate productive, antigen-specific GC responses. Accordingly, understanding the regulatory balancing that Tfr cells play in maintenance of B-cell tolerance while optimizing productive humoral immunity is crucial for vaccine-design strategies. To this end, we discuss recent evidence that Tfr cells promote humoral immunity and memory following viral infections, fitting with the accepted role of Treg cells in maintaining homeostasis with promotion of productive immunity, while mitigating that which is potentially pathological. We also propose models in which Tfr cells regulate antigen-specific B cell responses.

Antibodies form the first line of defense against invading pathogens and provide the basis for successful vaccines *via* humoral memory (1). Upon encounter with pathogens, naïve follicular B cells are activated and migrate into the T-B border of the spleen or interfollicular regions of lymph nodes, where they become fully activated upon further interaction with antigen-specific T cells (2–6). A subset of the activated B cells differentiates into short-lived plasmablasts secreting low-affinity antibodies in the splenic red pulp or medullary cords of lymph nodes, while in parallel, other B cells migrate back into follicle to seed early germinal centers (GCs) (7).

A subset of effector CD4^+^ T cells, T follicular helper (Tfh) cells defined by expression of the transcriptional factor Bcl6, also migrate into the early GCs. Therein, B cells undergo proliferation and somatic hypermutation of their immunoglobulin (Ig) genes, followed by a process in which the fittest B cells – those able to capture antigen *via* surface Ig and best present it on surface MHCII – are selected by follicular helper T (Tfh) cells (8). The strength of interaction between Tfh cells and GC B cells, which is proportional to the amount of antigen presented by GC B cells, drives the cyclic reentry and determines the cell cycle speed and number of division of GC B cell clones (9–11). The selected GC B cell clones then differentiate and mature into memory B cells and long-lived antibody-producing plasma cells, together forming the basis of humoral memory and associated pathogen protection. Upon pathogen re-exposure, high-affinity, protective antibodies secreted by LLPCs are a first line of protection; additionally, pathogen-specific memory B cells are activated and rapidly mature into plasmablasts to produce protective antibodies (12).

T follicular regulatory cells, or Tfr cells, express the germinal center (GC)-defining transcription factor Bcl6, and migrate to the B-cell follicle following immunization and infection, adding to the complexity of interplay between different cell types within the B cell follicle and GC and the heterogeneity of Treg cells that express T helper (Th) cell transcription factors (13–18). The localization of Tfr cells to the GC is believed to be dependent on the chemokine receptor CXCR5; however, a recent study showed that Tfr cells can access the GC in a CXCR5-independent manner, suggesting the possibility of other molecules, such as CXCR4 or S1PR2, playing a similar or redundant role of facilitating their migration (19). It is crucial to understand whether these Treg cells have acquired specialized function to regulate B cells responses and fine-tune their output, including development of humoral memory. Such insights will contribute to efforts in vaccine design.

Tfr cells differentiate from CD25^hi^ Foxp3^+^ Treg precursors (13, 20), while co-opting the developmental pathway of Tfh cells, dependent on the same signaling cues including CD28, signaling lymphocytic activation molecule (SLAM)-associated protein

(SAP), and inducible T cell co-stimulator (ICOS) (13, 14). One important aspect of the Tfr cell differentiation process is downregulation of CD25 expression (20–22), with these cells comprising both CD25^+^ and CD25^-^ populations with the former a transitional phenotype potentially because of their high CCR7 expression compared to their CD25^-^ counterparts (21). The necessity of downregulation of CD25 in Tfr cells lies in that IL-2 signaling leads to phosphorylation of Stat5 and the downstream upregulation of Blimp1, which antagonizes Bcl6 (23, 24). Thus, in order to upregulate Bcl6 expression, Tfr cells downregulate CD25, possibly through a mechanism of upregulation of ASCL2 (21, 25). Yet, the expression of key functional molecules in Tfr cells, such as Foxp3 and CTLA-4, are similar between CD25^+^ and CD25^-^ populations (21).

Treg cells are crucial for the maintenance of tolerance and homeostasis. Seminal work from Shimon Sakaguchi showed that athymic nude mice lacking T cells developed autoimmunity in several organs upon adoptive transfer of T cells lacking the CD4^+^CD25^+^ Treg population (26). This work demonstrated the classical function of Treg cells, which is limiting inflammation mediated by T effector cells to minimize associated tissue damage and prevent autoimmunity. Since then, various mechanisms of action by which Treg cells function in maintenance of immune and tissue homeostasis have been elucidated, which have been extensively reviewed elsewhere (27–30).

Accumulating evidence suggests that Treg cells also actively promote productive immune responses upon immune challenge, rather than functioning merely as suppressors. Early after infection, Treg cells optimize the chemokine milieu to ensure recruitment of the appropriate effector cells to sites of infection, for example in a model of mucosal herpes simplex virus infection (31). By controlling the overproduction of chemokines (CCL-2/3/4/5) from antigen-presenting dendritic cells (DCs), Treg cells also promote the avidity of CD8^+^ T cell primary responses by limiting priming of low-avidity CD8^+^ T cells (32–34). Subsequently, the disruption of high-avidity CD8^+^ T cell responses during the primary infection leads to an impaired CD8^+^ memory response in a model of recombinant *Listeria monocytogenes* infection (32). Treg cells have been found to act as an IL-2 sink during priming of CD8^+^ T cells to shield them from excessive IL-2 signaling, therefore favoring their differentiation into memory precursor effector cells rather than short-lived effector cells, thereby promoting effective secondary CD8^+^ T cell responses (35, 36). In a like vein, Treg cell-mediated dampening of inflammation during the resolution phase of the infection is crucial for the maturation of CD8^+^ T cell memory responses, mediated by Treg cell–derived IL-10 and CTLA-4 (37, 38). Treg cells additionally promote the formation of resident CD8^+^ memory T cells through the production of TGF-β in a model of West Nile virus infection (39). Independent from their role in suppression, Treg cells support tissue protection in an influenza virus infection model (40), reminiscent of their tissue repair function in nonlymphoid tissues (41). These lines of evidence, among others, support the idea that Tregs are essential for development of protective immune responses.

In line with these findings, we propose that functions of Tfr cells can be categorized into maintaining self-tolerance as well as promoting effective humoral responses. There are necessary mechanisms to maintain self-tolerance as B cells mature to antibody-secreting cells (ASCs) or memory cells because of the propensity for development of self-reactivity, particularly within the GC due to rapid proliferation with somatic hypermutation (SHM) of the antigen-binding variable region of immunoglobulin genes (42). Tfr cells thus serve as an additional level of regulation. For example, in the absence of Tfr cells, there is development of self-reactive ASCs in models of influenza virus infection, suggesting that one of the functions of Tfr cells is to prevent the expansion of autoreactive B cell clones (20). Protein immunization also revealed autoreactive IgG and IgE in the serum of mice lacking Tfr cells (43). Tfr-deficient mice also spontaneously develop autoantibodies at older age (44, 45). The mode of action by which Tfr cells maintain tolerance is incompletely understood, with CTLA-4 being implicated (46, 47) based on known function of this molecule in regulating peripheral tolerance (48) and with neuritin produced by Tfr cells suppressing the development of autoantibodies and IgE class switching (45).

Whether and how Tfr cells optimize GC responses and promote effective humoral memory is incompletely understood. In an protein immunization model, adoptive transfers of CXCR5-deficient Treg cells and naïve wild type CD4^+^ T cells into T-cell deficient recipients lead to enhanced antigen-specific antibody responses (14, 15). In genetically engineered mouse models lacking Tfr cells, antigen-specific B cell responses are elevated both *in vitro* and *in vivo* following immunization with protein-hapten conjugates and in a house dust mite challenge allergy model (43, 49), with other work revealing reduced antigen-specific IgE and IgG titers in Tfr-deficient mice in food allergen sensitization and immunization settings, respectively (50, 51). Different allergy models with distinct response kinetics and different Tfr-deficient animals were used in these two settings, which may potentially explain the contrasting results. In addition, depleting Tfr cells but not Tregs specifically using SAP KO (which do not form Tfh or Tfr cells but do form Treg cells) and Foxp3-DTR (which lack Tfr cells) mixed bone marrow chimeras as well as Bcl6^flox/flox^ Foxp3-Cre animals following a similar protein immunization model resulted in decreased antigen-specific GC B cells (13, 45).

Yet, it is likely that the function of Tfr cells depends on the complexity of the antigen, and its potential for replication. The maintenance of diverse antigen-specific and non-antigen-specific B cell clones upon viral infection in which the antigen is complex with distinct epitopes and replicative persistence is distinct from an immunization model with simpler, non-replicative antigens (52). The complexity of the antigen determines the kinetics of competition among different clones within the GC and therefore the rate of achieving homogenizing selection (52, 53). It is possible that one of the functions of Tfr cells is to maintain the breath of the response as it allows more layers of regulation in the selection process. Such function would be more important when there are lots of competing clones and there is the need of maintaining non-immunodominant antibody clones as in the case of influenza virus infection. Equally likely is the possibility that different infection or immunization routes in distinct local immune microenvironments leads to distinct Tfr cell-mediated effects (*e.*g., mucosal *versus* systemic). Consistent with the latter is the finding that mice developed impaired antigen-specific antibody response in the lack of polyclonal Treg cells in a mucosal immunization model (54), with the idea that Tfr cells contributed to the observed phenotype.

Recent studies have used influenza virus infection models to study the function of Tfr cells (20, 44). The total numbers of Tfh cells and GC B cells were not affected in the absence of Tfr cells at either day 9 post infection (p.i.) (44) or day 30 p.i (20). At day 9 p.i., the difference of anti-influenza virus antibodies in mice with and without Tfr cells was not detectable, although there was enhanced immune protection in the absence of Tfr cells (44). At day 30 p.i., there was a trend of reduced influenza virus-specific antibodies in the Tfr-deficient animals compared to controls (20). This work pointed to the potential of Tfr cells in regulating viral-specific GC output and humoral memory.

Our work has demonstrated that Tfr-cell derived IL-10 driving GC B cell transcriptional programs was necessary to maintain B cell responses following acute infection with lymphocytic choriomeningitis virus (LCMV), exemplifying the role of Tfr cells in optimizing productive humoral immunity following pathogen challenge (55). We subsequently showed that Tfr cells promote antigen-specific germinal center B cell responses during the late course of intranasal influenza virus infection (56). In the absence of Tfr cells, using Bcl6^flox/flox^ Foxp3-Cre animals, we observed alterations in the BCR repertoire, reduced numbers of virus-specific, long-lived plasma cells, as well as decreased antibody titers against both hemagglutinin (HA) and neuraminidase (NA), the two major influenza virus glycoproteins. To further investigate the functional relevance of Tfr cells during viral challenge, we utilized a sequential immunization model with repeated exposure of antigenically partially conserved strains of influenza viruses, revealing that Tfr cells promote recall antibody responses against the conserved HA stalk region. Thus, our studies in aggregate demonstrated that Tfr cells promote antigen-specific B cell responses and are essential for the development of effective long-term humoral memory.

These findings suggest that Tfr cells are necessary and sufficient to maintain the optimal antigen-specific humoral response, although the mechanism is unclear. How might Tfr cells necessarily suppress non-antigen specific or autoreactive GC B cells while sparing the antigen-specific ones? First, it is a model reminiscent of Treg cells optimizing CD8^+^ T cell responses with Treg cells preferentially suppressing responses by T cells that have weak, lower-affinity interactions with their cognate antigen (also the reason that Treg cells control self-reactive T cells due to their low-affinity) while sparing high-affinity interactions (32). One can imagine a similar scenario for the selection process of antigen-specific B cells regulated by Tfr cells within the GC. Specifically, as a positive selection signal is directly proportional to the strength of interaction between Tfh cells and GC B cells, Tfr cells selectively inhibit non-antigen-specific and autoreactive GC B cells based on their low-strength interactions with Tfh cells, whereas antigen-specific GC B cells that engage in high-strength interactions with Tfh cells overcome this suppression (**Figure 1**). The expression of positive selection signal, such as mTORC and cMyc signaling which are upregulated in GC B cells having recently received Tfh cell help, is likely to serve as a proxy for the strength of interaction that can be regulated by Tfr cells (57, 58). Furthermore, over-proliferation and outgrowth of non-antigen specific B cell clones in the absence of Tfr cells can compete for limited Tfh cell help within a given GC, therefore resulting in impaired antigen-specific B cell responses.

**Figure 1:**
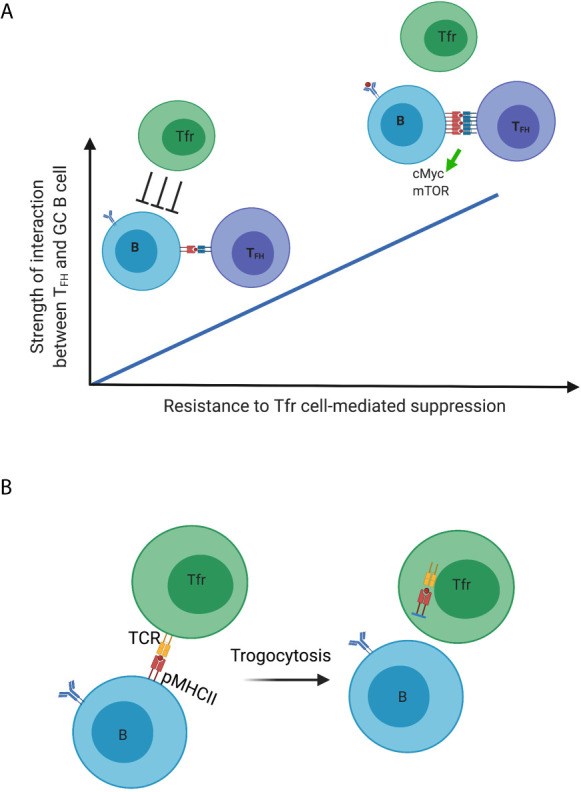
Dynamic regulation of germinal center B cells mediated by Tfr cells. **(A)** Model 1; **(B)** Model 2. Created with BioRender.com.

A second model by which Tfr cells promote productive immunity is their capacity to employ trogocytosis to reduce peptide-MHCII complexes on the surface of GC B cells, in this case those that are not specific to the immunizing antigen (**Figure 1**). Treg cells have been demonstrated to deplete peptide-MHCII complex from dendritic cells through trogocytosis in an antigen-specific manner to limit antigen presentation and subsequent effective priming of naïve antigen-specific T cells (59). Treg cells can also deplete CD80 and CD86 molecules on DCs in a similar fashion (60). If the specificity for Tfr cells is for self, and distinct from that of Tfh cells, the former may strip these antigens presented by GC B cells. With reduced antigen presentation, such GC B cells lose in the competition to other GC B cells presenting the immunizing antigen to elicit Tfh cell help. This would be another level of control to inhibit the outgrowth of autoreactive or non-antigen specific B cell clones. Our unpublished data show that Tfr cells indeed have higher surface expression of MHCII compared to that of non-follicular Treg cells at late time points following influenza virus infection (when the DC-Treg interaction is past the peak), which suggests the possibility of Tfr cells acquiring these molecules from the surface of GC B cells.

This second model is based on an assumption requiring further testing, that Tfr cells do not have specificity for the antigens of immune challenge or that the specificity for these is low. Treg cells can directly kill B cells through perforin and granzyme B in an antigen-specific manner (61); however, the specificity of Tfr cells is not well defined. There are contrasting results with work suggesting that Tfr cells can be specific for the immunizing antigen and the other data arguing that Tfr cells do not recognize the immunizing antigen with their repertoire distinct from that of Tfh cells which are antigen-specific (62, 63). One potential explanation for the distinct results is the differences in immunization models and immunizing antigens. Thus, it is important to examine the TCR specificity of Tfr cells in models using complex antigens in physiologically relevant models, such as influenza virus infection. Tfr cells differentiate from Treg cell precursors (13–15), so it is not surprising that there is substantial resemblance between the TCR repertoires of Tfr and Treg cells (63). However, it is not clear whether Treg cells recruited into the GC have specific TCR usage profile compared to the non-follicular Treg cells. The TCR repertoire analysis of Tfr cells following infection will answer questions of whether a particular subset of Treg precursors preferentially differentiate into Tfr cells and the polyclonality of these cells following influenza virus infection.

It will be of interest to identify Tfr-cell associated molecules (*e.g*., cytokines, surface molecules) that might mediate the aforementioned proposed processes of optimizing antigen-specific B cell responses within the GC. Furthermore, it is important to study the interaction among Tfr cells, GC B cells and Tfh cells to better understand the mechanism by which Tfr cells regulate the selection process of GC B cells. While our work and that of others demonstrate that Tfr cells do not affect Tfh cell numbers during infection (44), there is also limited evidence indicating that Tfr cells directly interact with GC B cells (45, 64). Imaging analyses are necessary to address how Tfr cells may choreograph the intricate dance within the GC. Interestingly, observations from us and others point to a population of Foxp3^+^ T cells in the follicles of lymphoid organs under steady state (65). It will be curious to explore whether these cells readily differentiate into Tfr cells and how they may regulate early responses in the follicles following infections.

## Author Contributions

Wrote the manuscript: YL and JC. All authors contributed to the article and approved the submitted version.

## Funding

This work was supported by grants from the NIH [R37AR40072 and R01AR074545 and from the Lupus Research Alliance (JC), a Yale Gruber Fellowship (YL), and a Yale Gershon Fellowship (YL)]. The authors declare no competing financial interests.

## Conflict of Interest

The authors declare that the research was conducted in the absence of any commercial or financial relationships that could be construed as a potential conflict of interest.
